# Mendelian randomization study confirms causal relationship between myopia and vitreous disorders

**DOI:** 10.1186/s12920-023-01673-x

**Published:** 2023-10-09

**Authors:** Jiayu Xu, Ya Mo

**Affiliations:** 1 Chengdu University of TCM, Sichuan, China; 2 Hospital of Chengdu University of TCM, Sichuan, China

**Keywords:** Myopia, Vitreous disorders, Mendelian randomization, Genome-wide association study

## Abstract

**Purpose:**

This study aims to investigate the potential bidirectional causal relationship between myopia and vitreous disorders from a genetic perspective, as vitreous disorders have been found to be closely associated with myopia development.

**Methods:**

To achieve this, a two-sample Mendelian randomization (MR) design was employed. The study utilized pooled statistics from independent genome-wide association studies. Myopia was chosen as the exposure factor, while five different vitreous disorders were considered as outcomes. The primary analytical method was the inverse variance weighting (IVW) method, supplemented by sensitivity analysis.

**Results:**

The study yielded significant findings indicating a positive association between myopia and vitreous disorders. The genetic prediction of myopia consistently demonstrated a positive correlation with vitreous disorders, as evidenced by IVW (odds ratio [OR] = 18.387; *P* < 0.01), MR Egger (OR = 2784.954; *P* < 0.01), weighted median (OR = 30.284; *P* < 0.01), and weighted mode (OR = 57.381; *P* < 0.01). All sensitivity analyses further validated these associations. Furthermore, a significant association was observed between myopia and other unspecified vitreous body disorders (IVW: OR = 57.729; *P* < 0.01).

**Conclusion:**

Studies mainly conducted in European populations have confirmed that myopia, extending beyond early high myopia, plays a crucial role in influencing vitreous disorders and that there is a unidirectional causal relationship between myopia and vitreous disorders. Additionally, a causal relationship was identified between myopia and other unspecified vitreous disordes. These findings introduce fresh perspectives for the clinical management of unspecified vitreous disorders and contribute to the understanding of the effect of myopia on vitreous disorders. Myopia prevention and treatment will aid in slowing down the process of vitreous liquefaction and subsequently decrease the incidence of malignant eye conditions.

## Introduction

Myopia is becoming the leading cause of visual impairment worldwide and is projected to affect approximately 500 million individuals by 2025, including a significant impact of high myopia on approximately 100 million people [1]. Family studies strongly support an important role for genetic factors in myopia. Specifically, the elongation of the eye’s axial length, a pivotal pathological myopia alteration, is mainly determined by heredity [2]. Heritability is as high as 94% [2]. Studies on myopia genetics have identified over 438 genetic markers linked to myopia and refractive errors, accounting for 18.4% of the overall heritability [3]. Despite these advances, a critical knowledge gap remains regarding the exact causal pathways involved.

Pathological changes in the vitreous of myopia have been well-documented. Axial myopia is associated with vitreous liquefaction, increased echo density, and deep vitreous degeneration [4]. In addition, elevated levels of proinflammatory cytokines and angiogenic growth factors have been identified in the vitreous fluid of myopic eyes [5]. Vitreous exosomal miR-143-3p and miR-145-5p have demonstrated potential as biomarkers for pathological myopia [6]. However, whether there are causal risk factors for these observed associations remains unclear. Mendelian randomization (MR) approaches are used to discern causal relationships between risk factors and outcomes from a genetic perspective [7–10]. MR findings often align with randomized controlled trials and provide evidence for validating drug targets [11]. Although this approach has been successful in many causal inference findings [12–14], there has been no discussion on the genetic perspective of myopia and vitreous diseases.

Our study employs a two-sample MR strategy to assess the potential causal link between myopia and different types of vitreous disorders. The assessment of these exposures and outcomes may hold implications for public health and clinical practices, enhancing myopia management and the prevention and early detection of the causes of vitreous disorders.

## Methods

### Study design

**Fig. 1:**
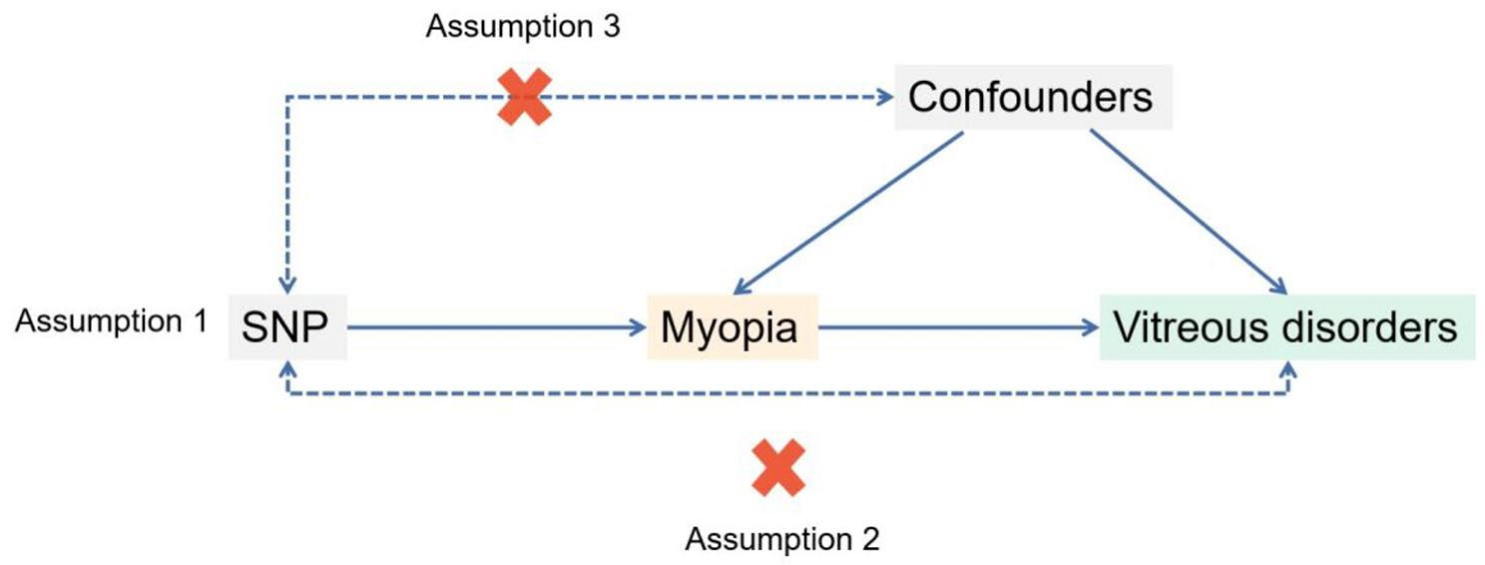
Diagram of MR principles and assumptions

This study employed a two-sample MR design to investigate the impact of myopia exposure on various outcomes using summary statistics from independent genome-wide association studies (GWAS). The outcomes examined were disorders of the vitreous body, crystalline deposits in the vitreous body, other vitreous opacities, vitreous haemorrhage, and other unspecified disorders of vitreous body. The MR design relies on three key assumptions concerning the genetic tool [15], namely, (1) its close association with the biomarker of interest, (2) its association with outcomes solely through exposure, without involving independent biological pathways, and (3) its lack of association with any confounding factors in the exposure-outcome relationship (Fig. 1).

### Summary statistics of myopia and vitreous disorders

Data for this study were sourced from The Medical Research Council Integrative Epidemiology Unit Open GWAS database (https://gwas.mrcieu.ac.uk/). The myopia dataset from GWAS (ukb-b-6353) was analyzed as the exposure factor, while data related to five vitreous diseases from the FinnGen database [16] were analyzed and employed as outcome factors. The primary outcome factor of interest was disorders of the vitreous body (finn-b-H7_ VITRBODYGLOBE).

Four additional vitreous diseases were explored in this study, namely, crystalline deposits in the vitreous body (finn-b-H7_ VITRCRYSTAL), other vitreous opacities (finn-b-H7_ VITROPACIT), vitreous haemorrhage (finn-b-H7_ VITRHAEMORR), and other unspecified disorders of the vitreous body (finn-b-H7_ VITROTH). For the MR analysis, only genetic variants with a genome-wide significance (*P* < 5 × 10^− 8^) were considered. The characteristics of each outcome factor are detailed in Table 1.

**Table 1:** Description of FinnGen Statistics for vitreous disorders

Trait	GWAS ID	Sample size (caose/control)	Number of SNPs	Population
Disorders of vitreous body	finn-b-H7_VITRBODYGLOBE	6,782/211,720	16,380,466	European
Crystalline deposits in vitreous body	finn-b-H7_VITRCRYSTAL	81/211,720	16,380,461	
Other vitreous opacities	finn-b-H7_VITROPACIT	453/211,720	16,380,461	
Vitreous haemorrhage	finn-b-H7_VITRHAEMORR	1,365/211,720	16,380,461	
Other and unspecified disorders of vitreous body	finn-b-H7_VITROTH	5,304/211,720	16,380,466	

All studies included in the open dataset received approval from respective institutional review boards. Written informed consent was obtained from all participants, who were exclusively of European ancestry.

### MR analysis and sensitivity analysis

MR analysis between exposure (myopia) and outcomes (vitreous diseases) was conducted using the TwoSampleMR v1.0.5 R package (R Foundation for Statistical Computing, Vienna, Austria) [17]. Genetic factors were chosen based on the following criteria: (1) myopic traits with *P* < 5 × 10^− 8^; (2) linkage disequilibrium r^2^ < 0.001; and (3) linkage disequilibrium distance > 10,000 kb. The primary method used was the inverse variance weighting (IVW) method [18], estimating the association between myopia and vitreous disorders. In addition, three other methods from the TwoSampleMR R package were employed: MR-Egger regression [19], weighted median method [20], and weighted mode method [17], accounting for potential horizontal pleiotropy. MR results were considered meaningful if the IVW method identified associations (*P* < 0.0083) and all four MR methods were effective in the same direction. To further assess the robustness of these identified associations, leave-one-out analyses were performed along with heterogeneity tests.

## MR results

### Myopia and disorders of vitreous body

**Fig. 2:**
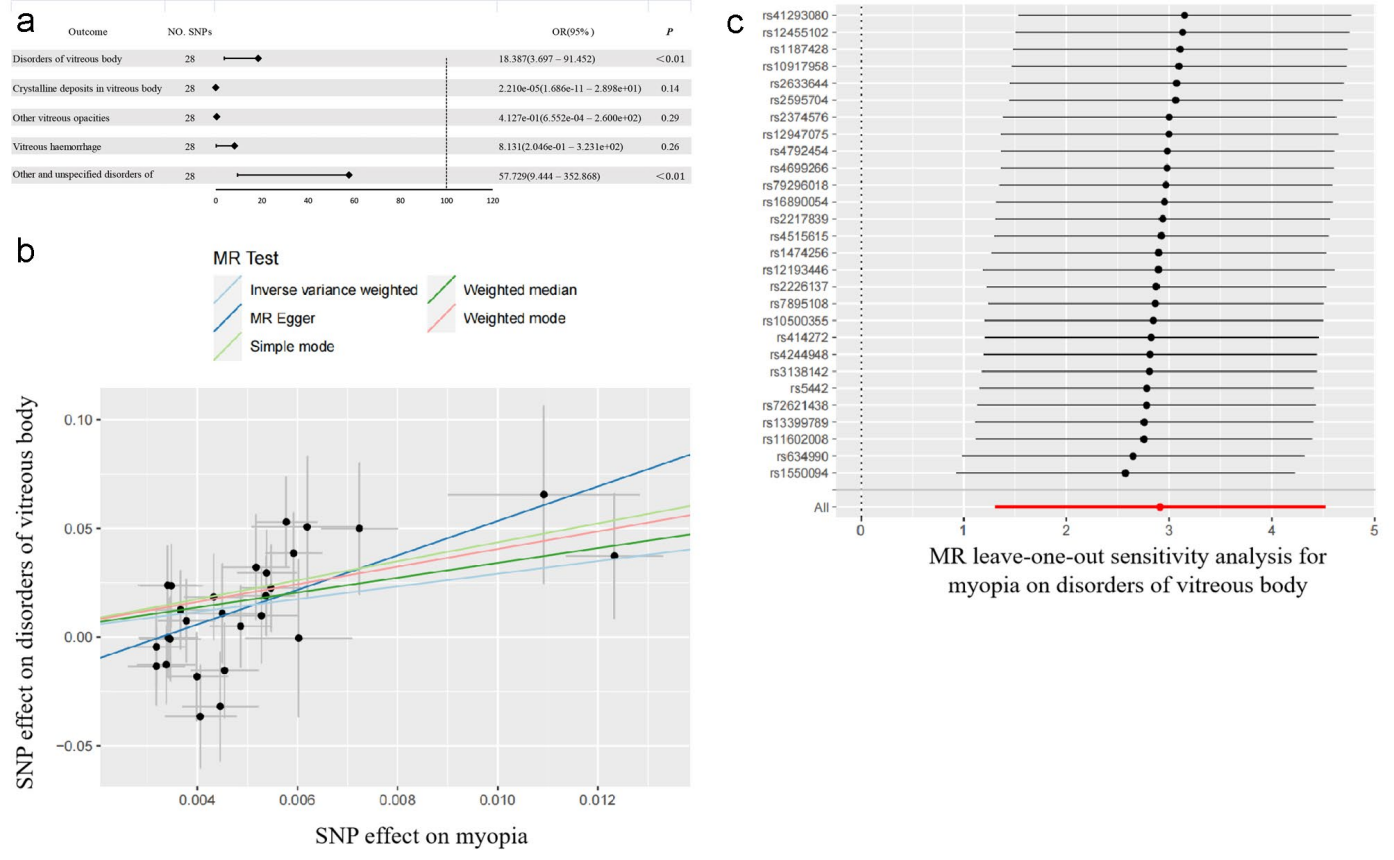
A MR forest plots of five vitreous disorders under the IVW. B Scatterplots for MR analyses of the causal effect of myopia on disorders of vitreous body. The slope of each line corresponds to the estimated MR effect per method. C Leave-one-out analysis of the causal effect of myopia on disorders of vitreous body. Each black point represents the IVW MR method applied to estimate the causal effect of adiponectin level on disorders of vitreous body, excluding that particular variant from the analysis. The red point represents the IVW estimate using all SNPs

Table 2 provides a summary of the MR analysis results. Examination of myopia and disorders of the vitreous body revealed a causal relationship between them (IVW: odds ratio [OR] = 18.387; 95% confidence interval [CI], 3.697–91.452; *P* < 0.01, MR Egger: OR = 2784.954; 95% CI, 20.974–369782.293; *P* < 0.01, weighted median: OR = 30.284; 95% CI, 2.795–328.135; *P* < 0.01, simple mode: OR = 78.031; 95% CI, 1.508–4038.495; *P* < 0.01, and weighted mode: OR = 57.381; 95% CI, 2.151–1530.879; *P* < 0.01; Fig. 2A). Figure 2B illustrates the scatter plot from the MR analysis, depicting the effect size of the association between myopia and vitreous diseases.

**Table 2:** Effect of myopia on disorders of vitreous body

Expousure	Outcome	SNP	method	OR	95% [CI]	*P*
Myopia	Disorders of vitreous body	28	MR Egger	2784.954	20.974-369782.293	**<0.01**
			Weighted median	30.284	3.022-303.521	**<0.01**
			Inverse variance weighted	18.387	3.697–91.452	**<0.01**
			Simple mode	78.031	0.962–6332.296	**<0.01**
			Weighted mode	57.381	1.825–1804.204	**<0.01**

To further verify the robustness of the causal relationship between myopia and disorders of the vitreous body, a sensitivity analysis was conducted. The leave-one-out analysis revealed no outliers (Fig. 2c). In addition, heterogeneity tests confirmed the absence of significant heterogeneity in both IVW and MR Egger models (*P* > 0.05). An inverse model was also tested, estimating the effect of disorders of vitreous body on the myopia. Notably, no association was noted (*P* = 0.66). Collectively, these findings confirm the existence of a causal relationship between myopia and vitreous diseases.

### Myopia and four other vitreous disorders

Table 3 presents the outcomes of the MR analysis. Strong associations were identified between myopia and other unspecified disorders of the vitreous body (IVW: OR = 57.729; 95% CI, 9.444–352.868; *P* < 0.01; MR Egger: OR = 18291.566; 95% CI, 73.495–4552465.865; *P* < 0.01; weighted median: OR = 150.033; 95% CI, 10.698–2104.119; *P* < 0.01; and weighted mode: OR = 563.954; 95% CI, 12.728–24987.776; *P* < 0.01). In contrast, no causal relationship emerged between myopia and the other three vitreous diseases (Fig. 2A).

**Table 3:** Effect of myopia Traits on other vitreous disordes

Expousure	Outcome	SNPs	Methods	OR	95% CI	*P*
Myopia	Crystalline deposits in vitreous body	28	MR Egger	1.282e + 02	3.670e-17–4.479e + 20	0.83
			Weighted median	1.256e-04	4.117e-13–3.831e + 04	0.37
			Inverse variance weighted	2.210e-05	1.686e-11–2.898e + 01	0.14
			Simple mode	6.986e-03	8.302e-18–5.879e + 12	0.78
			Weighted mode	2.165e-03	8.185e-15–5.724e + 08	0.65
	Other vitreous opacities	28	MR Egger	3.414e-01	7.299e-10–1.597e + 08	0.92
			Weighted median	8.349e-03	1.389e-06–5.018e + 01	0.28
			Inverse variance weighted	4.127e-01	6.552e-04–2.600e + 02	0.79
			Simple mode	3.355e + 02	3.964e-05–2.839e + 09	0.48
			Weighted mode	2.693e-03	5.600e-09–1.295e + 03	0.38
	Vitreous haemorrhage	28	MR Egger	145.078	1.664e-03–1.265e + 07	0.40
			Weighted median	1.589	8.357e-03–3.021e + 02	0.86
			Inverse variance weighted	8.131	2.046e-01–3.231e + 02	0.26
			Simple mode	0.719	1.164e-05–4.448e + 04	0.95
			Weighted mode	0.719	9.172e-05–5.644e + 03	0.94
	Other and unspecified disorders of vitreous body	28	MR Egger	18291.566	73.495–4552465.865	**< 0.01**
			Weighted median	150.033	9.709–2318.399	**< 0.01**
			Inverse variance weighted	57.729	9.444–352.868	**< 0.01**
			Simple mode	972.333	4.167–226879.121	**< 0.01**
			Weighted mode	563.954	15.929 –19966.053	**< 0.01**

**Fig. 3:**
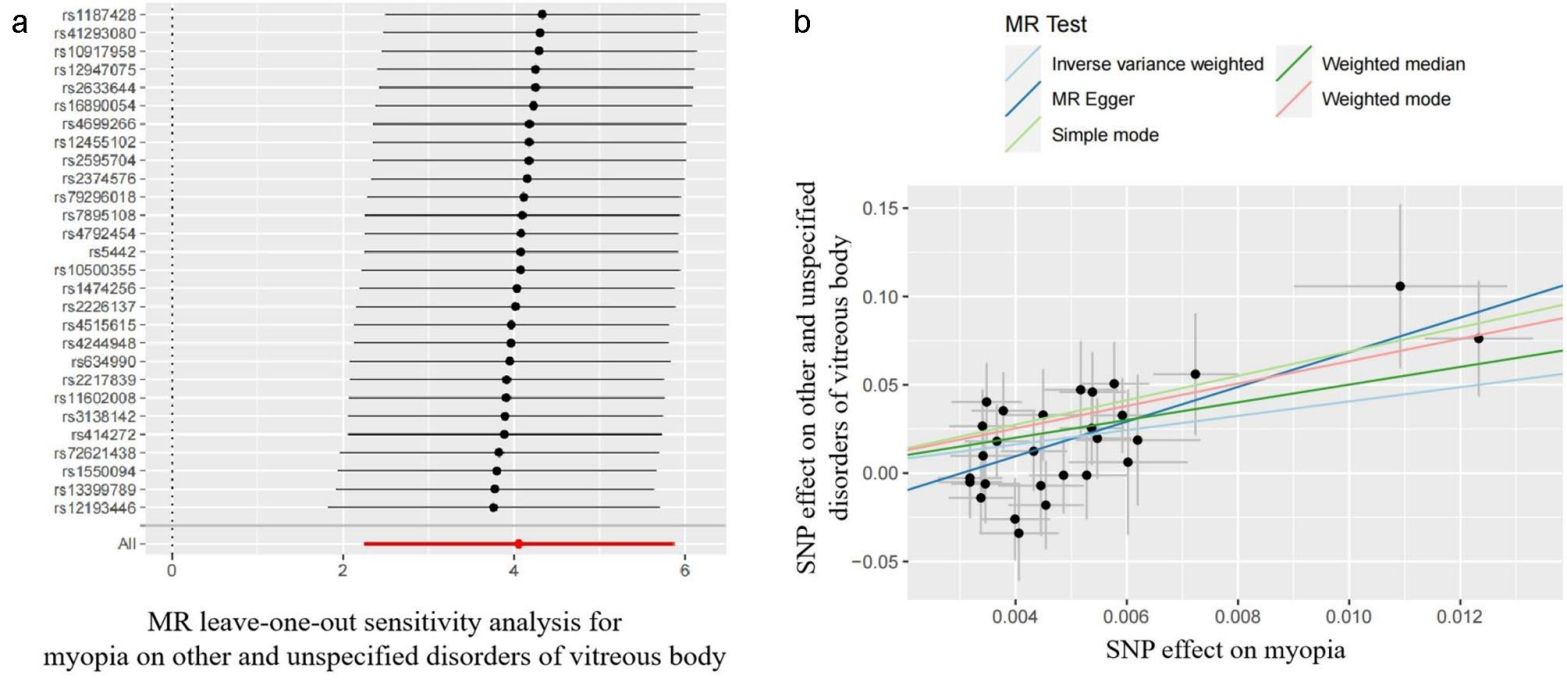
** A** Leave-one-out analysis of the causal effect of myopia on other and unspecified disorders of vitreous body. **B** Scatterplots for MR analyses of the causal effect of myopia on other and unspecified disorders of vitreous body

To ensure the stability of our results, we conducted a leave-one-out analysis for this specific analysis (Fig. 3A), and the results were consistent, with no outliers found. Figure 3B shows the scatter plot from the MR analysis, illustrating the effect size of the association between myopia and vitreous disorders.

## Discussion

This study used the MR framework to investigate the association between myopia and different types of vitreous diseases in a European population. Our findings provide genetic evidence for a plausible causal impact of myopia on the risk of disorders of the vitreous body. In particular, our analysis also found genetic evidence for a potential causal relationship between myopia and the risk of other unspecified disorders of vitreous body. These identified associations were found to be strong in sensitivity analyses, which addressed significant heterogeneity between genetic instrument effects. In contrast, we observed no evidence for genetic determinants of myopia associated with the risk of crystalline deposits in the vitreous body, other vitreous opacities, or vitreous haemorrhage within the European population.

Our results are consistent with those of prior observational studies that identified myopia as a risk factor for disorders of the vitreous body [4, 21]. Myopia is characterized by the presence of vitreous humor, which is caused by molecular changes that separate hyaluronic acid from collagen, leading to vitreous fiber liquefaction [22, 23]. As myopia advances, vitreous structure becomes more heterogeneous, forming small liquefied pockets called lacunae, while collagen fibers aggregate into larger, clinically opaque fibers, leading to a phenomenon termed visual “flying away” [24, 25]. In addition to causing structural heterogeneity, fibrous vitreous liquefaction can lead to posterior vitreous detachment, a clinically significant symptom known as “degenerative myopic blindness” [26]. These changes occur early in the lives of myopic individuals and are correlated with the degree of axial myopia [4, 27].

Utilizing MR methods, we obtained genetic evidence linking myopia with an increased risk of vitreous disorders. While the precise mechanisms behind this association remain partially understood, prior studies have proposed plausible biological explanations. The first mechanism involves retinal pigment epithelium damage impacting the vitreous, leading to reduced protein, collagen, and hyaluronic acid concentrations within the myopic vitreous [26]. Mouse models have also exhibited lowered levels of vital constituents such as potassium, sodium, chlorine, and proteins within the vitreous, which are essential for ocular tissue homeostasis and repair [28]. The second mechanism involves upregulation of oxidative stress and lipid metabolic pathways, thereby promoting vitreous liquefaction [29]. A recent study by Peng [30] reported a strong positive correlation between axial length and Dickkopf 1 levels in the vitreous. However, it is unclear whether this correlation is causal or consequent. Moreover, myopic eyes exhibit roughly 50% higher matrix metalloproteinase levels in the vitreous than in the control group, suggesting that the liquid vitreous in myopic eyes may be caused by enzymatic degradation of the existing gel-like vitreous [31].

Our study also revealed that genetically determined myopia increases the risk of other unspecified vitreous disorders among individuals of European descent. A comprehensive literature search revealed that other unspecified diseases of the vitreous body have not yet been clearly identified. This finding undoubtedly introduces novel perspectives for clinical exploration.

However, our analysis did not uncover a potential causal relationship between myopia and the occurrence of crystalline deposits in the vitreous body, vitreous opacities, or vitreous hemorrhage. Vitreous changes often remain unexplored in scientific, clinical, and economic literature concerning myopia [32]. Due to inadequate clinical and genetic evidence, the relationship between them has been poorly understood, likely because vitreous diseases are frequently neglected and understudied. Future MR studies leveraging more genetic instruments and larger samples may shed light on myopia and its potential impact.

This study is based on large-scale datasets evaluated through MR methods, which are less likely to be affected by confounding factors and reverse causality bias compared to conventional study approaches. Nonetheless, we should acknowledge several limitations. First, participants were exclusively of European descent, necessitating further research for generalizing findings to other ethnic groups. Second, the study adopted a cross-sectional design, underscoring the importance of alternative study designs to obtain the overall understanding of the impact of myopia on vitreous disorders. Lastly, there are few experimental studies on the impact of myopia on the incidence of vitreous disorders; therefore, further improvements in experimental designs and detection capabilities are necessary. Nevertheless, this study demonstrates that myopia progression poses a risk for vitreous disorders, which is an important factor affecting retinal health.

## Conclusion

Our study provides compelling genetic evidence that establishes a causal association between myopia and vitreous disorders in European populations. Furthermore, we confirm the causal relationship between myopia and other unspecified vitreous diseases from a genetic perspective. These findings carry substantial implications for advancing clinical diagnosis and treatment of vitreous disorders. By enhancing our understanding of vitreous diseases from a genetic perspective, we can mitigate the threat of myopia progression to the vitreous and reduce the occurrence of malignant events stemming from vitreous degeneration.

## Data Availability

The datasets generated and analysed during the current study are available in the IEU open gwas project [https://gwas.mrcieu.ac.uk/], and the GWAS ID are ukb-b-6353, finn-b-H7_ VITRBODYGLOBE, finn-b-H7_ VITRCRYSTAL, finn-b-H7_ VITROPACIT, finn-b-H7_ VITRHAEMORR, finn-b-H7_ VITROTH respectively.
